# Interventions to modify the habituation of biological responses to repeated stress in healthy adults: a randomized controlled trial

**DOI:** 10.1186/s13063-024-08620-w

**Published:** 2024-11-20

**Authors:** Johanna Janson-Schmitt, Nicolas Rohleder

**Affiliations:** Department of Psychology, Friedrich-Alexander-Universität Erlangen-Nürnberg, Nägelsbachstraße 49a, Erlangen, 91052 Germany

**Keywords:** Rumination, Self-compassion, Repeated stress, Inflammation, Gene expression, Habituation, Allostatic load, Stress response patterns

## Abstract

**Background:**

Changes in response patterns of biological stress systems, including responses of the sympathetic nervous system (SNS) and the hypothalamus–pituitary–adrenal (HPA) axis to repeated stress, can promote the development and progression of chronic diseases via changes in downstream inflammatory processes. The aim of this project is thus to investigate, whether habituation of biological stress system activity including responses of the inflammatory system can be modified. Aiming to test for possible paths of action, a randomized controlled study with two intervention programs designed to manipulate cognitive coping strategies will be carried out. By increasing either ruminative or self-compassionate thoughts among healthy young adults, the intervention programs are expected to affect the regulation of occurring emotions as expressed by the responsiveness of biological systems during repeated stress exposure.

**Methods:**

In this study, a total of 120 healthy adults will complete the Trier Social Stress Test (TSST) on two consecutive days. Immediately after the first stress induction, participants will be randomly assigned to two experimental conditions designed to manipulate cognitive coping strategies (rumination vs. self-compassion) or a control condition. Measures of HPA axis (salivary cortisol) and autonomic activity (salivary alpha amylase, heart rate, heart rate variability) as well as inflammatory markers (plasma interleukin(IL)-6, expression rates of pro- and anti-inflammatory cytokine genes) will be repeatedly assessed throughout the experimental sessions. Response and habituation indices of these measures will be calculated and compared between the experimental conditions and the control condition.

**Discussion:**

The results should provide insight into whether modifying response patterns of biological stress systems could reverse a significant biological mechanism in the development of stress-related diseases.

**Trial registration:**

German Clinical Trials Register (DKRS), DRKS00034790. Registered on August 12, 2024, https://www.drks.de/DRKS00034790.

**Supplementary Information:**

The online version contains supplementary material available at 10.1186/s13063-024-08620-w.

## Introduction

### Background

Psychological stress experience has become a serious issue in modern society and is a major cause of physical health impairments. The impact of psychological stress is also gaining significant importance in the workplace: as exemplified by German health insurance companies, the number of days absent due to mental disorders increased by 48.4% between 2012 and 2022 [1], resulting in costs amounting to billions for companies and the economy. In recent years, German healthcare costs of mental illnesses amounted to 56.4 billion euros in 2020 [2] and 15.8 billion euros in lost production costs were recorded in 2021 [3]. However, since mental disorders, such as frequent and chronic stress experience, also contribute to the development of numerous somatic diseases [e.g., cardiovascular diseases, 4], it is expected that the negative economic consequences may even aggravate due to long-term effects of psychological stress on physical health. Counteracting these effects in the long-term by designing effective health-promoting intervention programs requires an understanding of underlying biological processes. Thus, the proposed project aims to investigate whether biological adaptation to repeated stress and associated pathophysiological processes can be modified.

Altered activity of biological stress systems, including the hypothalamus–pituitary–adrenal (HPA) axis, the sympathetic nervous system (SNS), and the inflammatory system, is considered to play a mediating role between stress experience and pathophysiological processes [5]. While an effective interplay of these systems and the resulting endocrine and inflammatory responses contribute to enhanced immune functions during acute stress exposure [6], dysregulations of these systems, caused for example by repeated or long-term stress experience, may lead to adverse health effects [7]. For instance, a derailment of the inflammatory system—a consequence of chronic stress experience—has been established as a significant contributor to the development of important non-communicable diseases such as cardiovascular diseases, metabolic diseases as well as some cancers [8, 9].

Within the framework of the allostatic load model [10, 11], which posits a causal relationship between maladaptive stress response patterns and the development of diseases, McEwen [7] describes the lack of habituation of biological stress responses to repeated stressors as a potential mechanism that plays a crucial role in disease progression. More specifically, non-habituation is considered a dysfunctional adaptation mechanism to repeated stressful events leading to wear and tear on the system over time and consequently to an accumulation of allostatic load. Conversely, biological responses being able to habituate to recurrent stressors (i.e., to adapt) are seen as a protective factor against the accumulation of allostatic load. Accordingly, the extent to which an individual’s biological stress response habituates to repeated stress is regarded as a determinant for long-term health [12].

A large number of studies has revealed associations between stronger habituation of biological stress system activity and various biopsychosocial determinants, including, for example, self-compassion [13], rumination [14], emotion regulation [15], gender [16], and resilience [17], whereas experimental research designed to increase habituation ability is scarce. For instance, Manigault et al. [18] attempted to modify HPA axis activity in response to repeated stress and reported increased HPA axis habituation in a population of healthy adults after being exposed to either a 6-week mindfulness-based stress reduction intervention (*n* = 31) or a 6-week cognitive-behavioral stress management intervention (*n* = 34). Similarly, Asbrand et al. [19] attempted to increase HPA axis habituation to repeated stress in socially phobic children (*n* = 32), demonstrating that, upon completion of 12 weeks of cognitive–behavioral stress management intervention, cortisol responses remained either robust (i.e., did not change in their intensity) or decreased over repeated stress exposure. As demonstrated by these experiments, psychotherapeutic intervention measures therefore prove to be an effective method for modification of biological stress system activity. Accordingly, cognitive–behavioral techniques (e.g., cognitive restructuring, relaxation, and self-regulation strategies) may facilitate cognitive reappraisal of anxiety-inducing stimuli and down-regulation of upcoming specific emotions during psychosocial stress, resulting in altered stress response patterns of HPA axis activity as expressed by higher HPA axis habituation to repeated stress [18].

### Summary and objective

In summary, habituation of biological stress system activity to repeated stress exposure is considered a protective mechanism counteracting the accumulation of allostatic load [12]. While determinants of biological stress response habituation have been studied in depth [e.g., 17], only few experimental studies have addressed the modifiability of biological stress response patterns by promoting biological stress response habituation [18, 19]. As shown by these studies, habituation of HPA axis activity to repeated stress can be successfully increased in both healthy individuals and socially phobic children using psychotherapeutic techniques [18, 19]. However, due to the scarcity of studies demonstrating that psychotherapeutic intervention programs may effectively modify habituation of biological stress system activity, it remains unclear whether stress response habituation of other health-relevant systems, including the SNS and in particular the inflammatory system, may be targeted using equivalent interventional approaches.

Following this line of reasoning, we developed a randomized controlled procedure which addresses the manipulation of coping strategies, which in turn are expected to affect the regulation of occurring emotions as expressed by the responsiveness of biological stress systems during repeated stress exposure. To investigate possible paths of action, the intervention program aims to manipulate two different coping strategies that have been shown to be either positively [self-compassion, 13] or negatively [rumination, 14] related to biological stress response habituation. Subsequently, we aim to test whether the manipulation of coping strategies causes changes in the habituation capacity of biological stress system activity. For this purpose, stress-induced changes of endocrine, autonomic, and inflammatory parameters will be assessed in order to determine the effects of psychological coping on biological stress response habituation and, in particular, the ability to modify stress-induced inflammatory response patterns. Overall, we anticipate self-compassion to promote and rumination to impede biological stress response habituation. Our specific hypotheses are specified in the “Methods” section below (see “Hypotheses”).

## Methods

### Study design

The study is designed as a randomized controlled, single-center, and single-blinded trial, in which participants will be randomly assigned to one of two intervention groups (rumination vs. self-compassion) or a control condition (see “Randomization”) with an allocation ratio of 1:1:1. The study employs a superiority trial design, aiming to determine whether one of the two cognitive coping interventions is more effective in modifying the responsiveness and habituation of biological stress systems compared to the control condition. The design encompasses repeated measurements of each group, i.e., each group will undergo two experimental sessions (t1 and t2) comprising stress test exposures, and within each experimental session, repeated biological and psychological outcome assessments will take place (see Table 1).

**Table 1:**
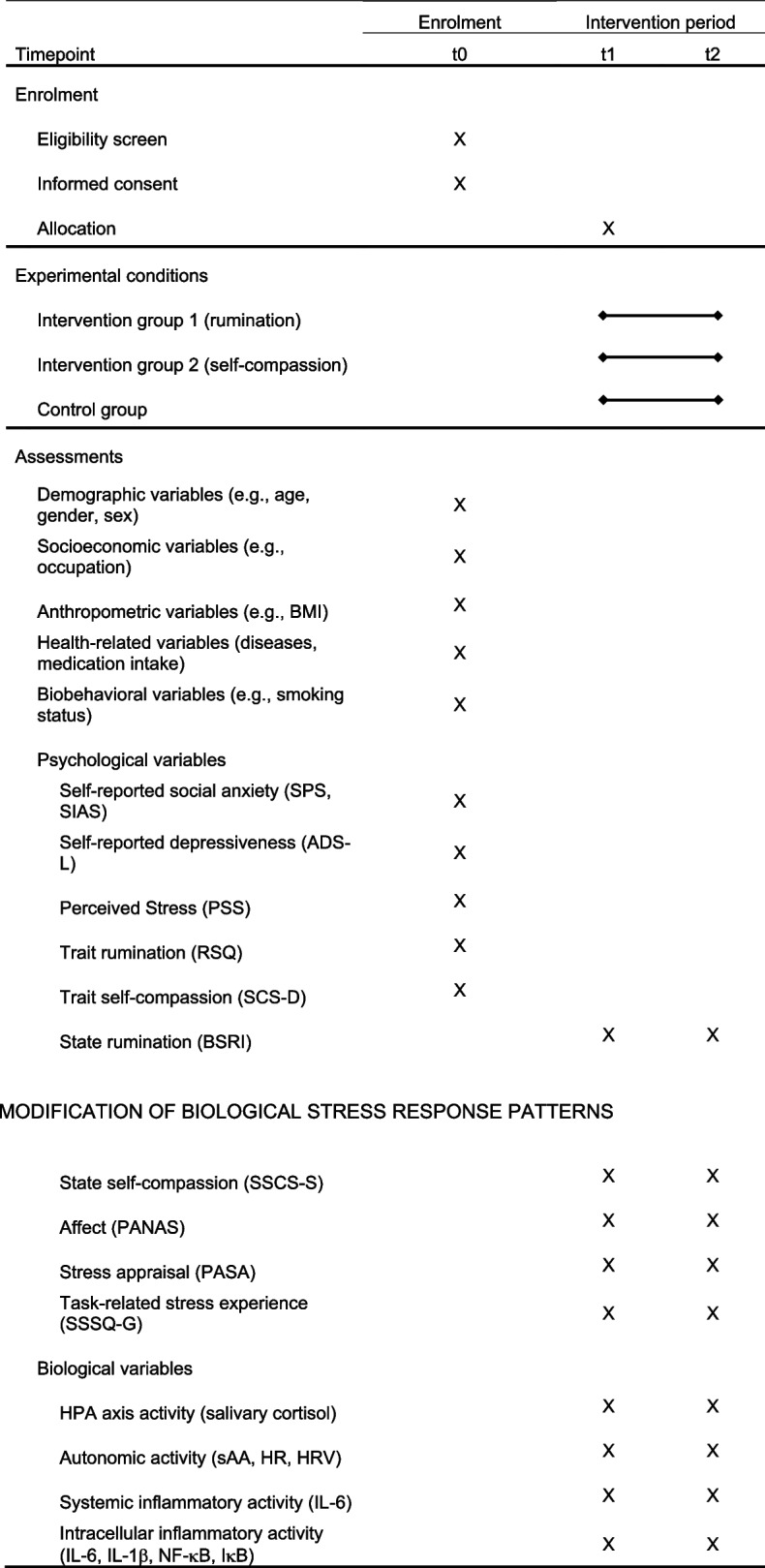
Study time line for the schedule of enrolment, experimental conditions, and assessments

*ADS-L* Allgemeine Depressionsskala Langform, *BMI* Body Mass Index, *BSRI* Brief State Rumination Inventory, *HPA axis* hypothalamus-pituitary adrenal axis, *HR* heart rate, *HRV* heart rate variability, *IкB* I kappa B kinase, *IL* Interleukin, *NF-кB* nuclear Factor kappa B, *PANAS* Positive and Negative Affect Schedule, *PASA* Primary Appraisal Secondary Appraisal, *PSS* Perceived Stress Scale, *SCS-D* Self Compassion Scale Deutsch, *RSQ* Response Style Questionnaire, *sAA* salivary alpha-amylase, *SPS* Social Phobia Scale, *SIAS* Social Interaction Anxiety Scale, *SSCS-S* State Self-Compassion Scale Short Form, *SSSQ-G* Short Stress State Questionnaire German

### Participants

A total of *N* = 120 (*n* = 40 per condition) community-dwelling adults from the Nürnberg/Fürth/Erlangen region (Bavaria, Germany) will be recruited via advertisements and offered monetary compensation (50 Euros) if included in the study. Before testing, participants receive information sheets via email and will have the possibility to contact study staff with any queries. Participants will then be screened for eligibility using EFS Survey Unipark (Tivian XI GmbH, Cologne, Germany), which will continue until the target population is achieved. Written informed consent will be obtained electronically from each participant at the beginning of the screening process.

**Fig. 1:**
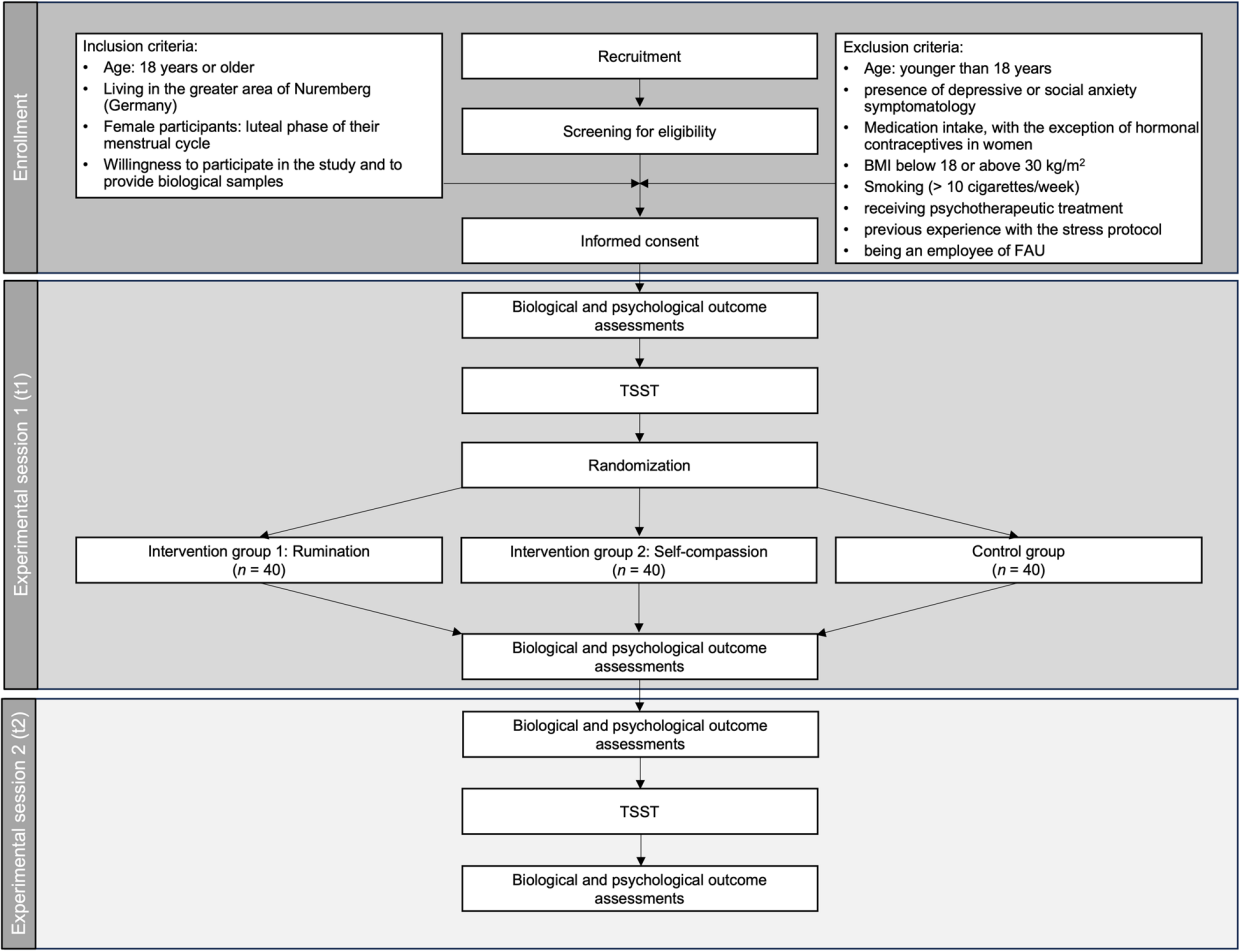
Flow diagram including the process of enrollment, allocation, interventions, and assessments

Eligible participants must be at least 18 years old and willing to participate in the study and provide biological samples. Female participants will be tested in the luteal phase of their menstrual cycle. Exclusion criteria are (1) younger than 18 years of age, (2) presence of depressive or social anxiety symptomatology, (3) presence of acute and/or chronic somatic diseases, (4) medication intake (e.g., beta blocker, glucocorticoids, anti-depressants), with the exception of hormonal contraceptives in women, (5) body mass index (BMI) below 18 or above 30 kg/m^2^, (6) smoking (> 10 cigarettes/week), (7) receiving psychotherapeutic treatment at study entry, (8) previous experience with the stress protocol, and (9) being an employee of Friedrich-Alexander-Universität Erlangen-Nürnberg (FAU). For an overview of the enrolment procedure, see Fig. 1.

### Ethics approval

The study will be conducted in accordance with the Declaration of Helsinki and has been approved by the Ethics Committee of the FAU’s Medical Faculty (protocol number 10_21 B). Protocol amendments will be communicated in writing to the Ethics Committee, as well as to other relevant parties, including the German Research Foundation (DFG) and the German Clinical Trials Register (DRKS).

### Power analysis

An a priori power analysis has been conducted using G*Power (version 3.1.9.6) for sample size estimation, based on the findings of a preliminary study [20]. The analysis is limited to the reference biomarkers cortisol and sAA, as no available data provide insights into the modifiability of inflammatory response patterns.

As demonstrated in the aforementioned preliminary study [20], the findings suggest a high modifiability of cortisol and sAA responses through the manipulation of psychological coping mechanisms. Specifically, a large effect was observed in the difference in cortisol response habituation between intervention and control group (*d* = 0.81), while a moderate-to-large effect was found for the difference in sAA response habituation (*d* = 0.67). Based on these findings, medium-to-large effects are expected for differences between experimental groups with regard to their difference in habituation of biological stress system activity. Power analyses revealed a sample size of *N* = 111 (*n* = 37), using an effect size of *f*^2^ = 0.30, *α* = 0.05, 1-*β* = 0.80, number of groups = 3. To address the anticipated 10% dropout rate caused by non-appearance of participants on study day 2 or insufficient biological sample volumes, an additional nine participants (three per condition) will be recruited and tested in this study, resulting in a total sample size of *N* = 120.

### Status and timeline of the study

Recruitment of participants will start in August 2024. Data collection will commence in September 2024 and continue until September 2026. Data analysis will be conducted upon completion of the entire data collection. No preliminary data analyses will be performed.

### Experimental conditions

Figure 1 provides an overview of the allocation of participants into three experimental conditions. Participants assigned to intervention group 1 will take part in a rumination intervention, whereas participants from intervention group 2 will take part in a self-compassion intervention. Condition 3 will serve as an active control group in which participants are instructed to think neutrally about a specified topic and record their thoughts in written form.

### Tasks and interventions

#### Intervention group 1: rumination

According to the Response Styles Theory [21], rumination is described as repetitive thought process focused on the causes, consequences, and symptoms of negative affect, which may generally cause maintenance and amplification of depressive symptoms. Furthermore, Clark and Wells [22] describe rumination that specifically occurs after psychosocially stressful events (post-event rumination) as persistent processing or reflection about a previous social interaction.

As has been revealed by previous research, rumination is generally associated with indicators of lower psychological well-being, as it predicts the occurrence of future depressive episodes [23]. Additionally, individuals who strongly ruminate about prior stressful events exhibit dysfunctional biological adaptation mechanisms, including higher cortisol responses to acute stress [24], and sustained activation of the HPA axis and the inflammatory system [25]. Preliminary work has also revealed associations between stressor-related rumination and increased stress-induced responses of HPA axis activity to repeated acute stress, suggesting that rumination may impair the habituation of biological stress system activity [14]. Due to the persistent and passive focus on negative emotions in stressful situations as well as the impaired ability to adequately biologically adapt to acute and repeated stress, rumination is thus considered to have detrimental effects on long-term health.

As demonstrated by multiple studies, rumination can be experimentally induced, which affects the activation of the HPA axis and the inflammatory system [25, 26]. An analysis with preliminary data reveals that stressor-related rumination can be induced by a short-term writing intervention which leads to a reduction of HPA axis habituation [20]. Analogous to this experimental approach, the planned study aims to manipulate rumination as coping mechanism to gain insight into whether it may lead to maladaptive biological adaptation. To this end, the first intervention is designed to experimentally induce rumination by instructing participants to ruminate about their first stress exposure and, in particular, about their negative feelings and to write down their thoughts.

#### Intervention group 2: Self-compassion

Self-compassion refers to a personality trait that describes an open and caring attitude towards oneself in the face of failure and perceived shortcomings. It is characterized by self-kindness, a perception of common humanity and mindfulness in dealing with negative thoughts and emotions [27–29]. Overall, research indicates that individuals reporting higher self-compassion are mentally and physically healthier [30]. Our own preliminary work has shown that individuals with higher self-compassion exhibit lower cortisol and salivary alpha-amylase (sAA) responses and thus healthier biological adaptation to acute stress [13]. Due to the increased stress resilience (i.e., the improved ability to experience negative emotions during stress, and to show adaptive biological stress responses), self-compassion is thus considered being protective with regard to long-term health.

As initially demonstrated by Neff and Germer [31], self-compassion can be enhanced through intervention. Thus, the Mindful Self Compassion program developed by Neff and Germer [31] demonstrably leads to a sustained increase in self-compassion and mindfulness and to a reduction in anxiety and depression. It has also been shown that the program leads to increases in overall well-being [32] and keeps blood glucose levels stable in diabetic patients [33]. Similarly, Diedrich et al. [34] presented a training designed to enhance self-compassion, which has been shown to reduce depressive mood in patients with major depressive disorder, making it an effective short-term intervention, at least on a psychological level. Due to its high efficiency, the procedure described by Diedrich et al. [34] will be applied in the present study in order to manipulate self-compassion. Therefore, participants from intervention group 2 will be instructed to think about their first stress exposure, to actively engage in self-compassion, and to write down their thoughts.

#### Control group

To ensure that any changes in stress responses are attributed to the effect of specific cognitive strategies (rumination vs. self-compassion), rather than to engaging in a thinking and writing task per se, a control group will be included in which participants will be instructed to think neutrally about a specified topic (e.g., a description of everyday life) and to write down their thoughts.

### Assessment of sample characteristics during screening

Demographic (e.g., age, gender, sex), socioeconomic (e.g., occupation), anthropometric (e.g., BMI), health-related (diseases, medication intake), and biobehavioral (e.g., smoking status) variables will be electronically assessed during screening and will partly be used to determine eligibility for participation (see “Participants”).

### Psychological assessments during screening

During the screening process, additional psychological variables (e.g., self-reported social anxiety) will be electronically assessed, which will partly be used to determine eligibility for participation (see “Participants”).

#### Self-reported social anxiety

To exclude altered response patterns of HPA axis activity in social anxiety disorders [35], the severity of symptoms will be assessed using the Social Phobia Scale [SPS, 36] and the Social Interaction Anxiety Scale [SIAS, 36]. Each scale comprises 20 items designed to identify situations where socially anxious individuals typically experience emotions of fear. The SPS assesses anxiety in performance situations (e.g., “I get tense when I speak in front of other people”), while the SIAS assesses anxiety in social interaction situations (e.g., “I have difficulty making eye contact with others.”). Three negatively phrased items of the SIAS will be reverse-coded, and total scores will be calculated by summing up items on each scale. The presence of a social anxiety disorder will be determined by comparing the scale scores to predefined cutoff values (SPS > 21, SIAS > 32).

#### Self-reported depressiveness

To exclude altered cortisol response patterns in depressive disorders [37], the severity of depressive symptoms will be assessed using a German version of the Center for Epidemiological Studies Depression Scale [CES-D, 38] termed “Allgemeine Depressionsskala Langform” (ADS-L) from Hautzinger et al. [39]. The 20-item scale determines the severity of depressive symptoms, by asking participants how often in the past week they experienced symptoms such as “I felt sad” or “My appetite was poor”. Four items will be reverse-coded, and total scores will be calculated by summing up all item scores. The presence of a depressive disorder will be determined by comparing the scale scores to a predefined cutoff value (> 22).

#### Perceived stress

To assess participants’ perceived stress, a German version of the 10-item Perceived Stress Scale [PSS, 40] from Klein et al. [41] will be used. In this questionnaire, participants will be asked to indicate how often they have experienced stress in the past month, for example, “In the last month, how often have you felt nervous and ‘stressed’?”. Ratings will be made on a 5-point Likert scale ranging from “never “ to “very often “. After reverse-coding four items, overall sum scores will be computed.

#### Rumination

Trait rumination will be assessed using a German version of the Response Style Questionnaire [RSQ, 42] from Kühner, Huffziger, and Nolen-Hoeksema [43]. The scale evaluates how participants typically respond to depressive mood and includes two subscales Rumination (21 items) and Distraction (11 items). Items on the Rumination scale depict reactions focusing on depressive mood, its causes, and consequences (e.g., “Think about how alone you feel”), while items on the Distraction scale describe active and problem-solving approaches to reduce feelings of depression (e.g., “Help someone else with something in order to distract yourself”). Ratings will be recorded on a 4-point Likert scale ranging from “almost never” to “almost always,” with nine negative items being reverse-coded. Subsequently, scores will be computed by summing up all items for each response style.

#### Self-compassion

To assess trait self-compassion, a German version of the Self Compassion Scale [SCS, 28] from Hupfeld and Ruffieux [SCS-D, 27] will be utilized. The scale comprises 12 items addressing self-kindness (e.g., “I try to be loving towards myself when I’m feeling emotional pain.”), self-judgment (e.g., “I’m disapproving and judgmental about my own flaws and inadequacies.”), perceptions of common humanity (e.g., “When things are going badly for me, I see the difficulties as part of life that everyone goes through.”), perceptions of isolation (e.g., “When I think about my inadequacies, it tends to make me feel more separate and cut off from the rest of the world.”), mindfulness (e.g., “When something upsets me I try to keep my emotions in balance.”), and over-identification (e.g., “When I’m feeling down, I tend to obsess and fixate on everything that’s wrong.”). Ratings will be made on a 5-point Likert scale ranging from “almost never” to “almost always”. Following reverse-coding of items assessing perceptions of self-judgment, isolation, and over-identification, total sum scores will be computed.

### Psychological assessments during the experiment

During each experimental session, i.e., before, during, and after completion of the Trier Social Stress Test (TSST), further electronic assessments of psychological variables will take place.

#### Rumination

Changes in ruminative thinking will be assessed repeatedly before and after completion of the TSST (i.e., at − 1 as well as + 20 min relative to the TSST) using a German version of the 8-item Brief State Rumination Inventory [BSRI, 44] from Michel-Kröhler, Wessa, and Berti [45]. In this questionnaire, participants will be instructed to indicate how they feel or think in the present moment. The scale comprises eight items (e.g., “Right now, I am reflecting about my mood.”) which will be answered on a 100-mm visual analog scale ranging from “completely disagree” (0) to “completely agree” (100). Total scores will be obtained by summing up all items. The translated version of the questionnaire has not been validated yet, however, among existing state rumination measures in English the BSRI is regarded as the most established and appropriate measure for the present study.

#### Self-compassion

To capture the current level of participants’ state self-compassion during the experimental sessions, we have translated the items of the State Self-Compassion Scale Short Form (SSCS-S) from Neff et al. [46] into German, which have not been validated yet. In this questionnaire, participants will be asked to indicate how they are feeling toward themselves in the present moment. All six statements of the SSCS-S (e.g., “I’m giving myself the caring and tenderness I need.”) will be answered on a 5-point Likert scale ranging from “not at all true for me” to “very true for me”. Three items will be reverse-coded, and total means will be calculated to obtain total state self-compassion scores. To assess changes in state self-compassion over the course of each experimental session, state self-compassion will be measured repeatedly before and after completion of the TSST (i.e., at − 1 as well as + 20 min relative to the TSST).

#### Affect

Participants’ affective states will be assessed using a German version of the Positive and Negative Affect Schedule [PANAS, 47] from Krohne et al. [48]. The scale comprises 20 items which ask participants to judge how strongly they experience positive (e.g., “excited”) or negative (e.g., “angry”) emotions during a specific task or situation. Ratings will be made on a 5-point Likert scale ranging from “not at all” to “very much”, and total scores will be calculated by summing up items on each subscale. To check whether the experimental manipulations were successful on a psychological level, affect will be measured repeatedly before and after completion of the TSST (i.e., at baseline, − 1 as well as + 1, + 20, + 120 min relative to the TSST).

#### Stress appraisal

During the preparation period of the TSST, participants’ anticipatory stress appraisal will be assessed using a German version of the 16-item Primary Appraisal Secondary Appraisal (PASA) questionnaire from Gaab [49]. The questionnaire was specifically designed to assess cognitive appraisal processes and measures participants’ anticipatory appraisals of challenge (e.g., “This task challenges me.”), threat (e.g., “This situation scares me.”), self-concept of own abilities (e.g., “In this situation, I know what I can do.”), and control expectancies (e.g., “I can best protect myself from failure in this job interview through my own behavior.”). Ratings will be made on a 6-point Likert scale ranging from “strongly disagree” to “strongly agree”, and after reverse-coding five items, final scores will be computed by summing up all item scores.

In addition, participants’ experience of task-related stress will be repeatedly assessed over the course of each experimental session using a German version of the 24-item Short Stress State Questionnaire [SSSQ, 50]—a short version of the Dundee Stress State Questionnaire [DSSQ, 51] from Ringgold et al. [SSSQ-G, 52]. In this questionnaire, participants will be asked to indicate how well each adjective describes how they feel before and during the task (e.g., “dissatisfied”) and how strongly they experience positive (e.g., “excited”) or negative (e.g., “angry”) emotions before and during the TSST. Ratings will be made on a 5-point Likert scale ranging from “not at all” to “extremely”, and total scores will be calculated by summing up items on each subscale. The questionnaire will be filled out before and after completion of the TSST (i.e., at − 1 as well as + 1 min relative to the TSST).

### Biological assessments during the experiment

Main outcomes represent measures of biological stress system activity, including HPA axis and autonomic activity, which will be assessed via saliva samples and continuous electrocardiogram (ECG) recordings, as well as stress-induced responses of the inflammatory system, which will be assessed via peripheral blood samples. Table 2 provides an overview of all biological outcome measures.

**Table 2:** Biological outcome measures

Biological outcome measure	Number of samples per session	Sampling time points per session	Sampling procedure
Autonomic nervous system			
sAA	7	Baseline, − 1, + 1, + 10, + 20, + 30, + 45 min	Saliva sample
HR	-	Continuously	ECG
HRV	-	Continuously	ECG
HPA axis			
Cortisol	7	Baseline, − 1, + 1, + 10, + 20, + 30, + 45 min	Saliva sample
Inflammatory system			
IL-6	3	− 1, + 30, + 90 min	Peripheral blood sample
IL-6 expression	3	− 1, + 30, + 90 min	Peripheral blood sample
IL-1β expression	3	− 1, + 30, + 90 min	Peripheral blood sample
NF-кB expression	3	− 1, + 30, + 90 min	Peripheral blood sample
IкB expression	3	− 1, + 30, + 90 min	Peripheral blood sample

*ECG* electrocardiogram, *HPA axis* hypothalamus-pituitary adrenal axis, *HR* heart rate, *HRV* heart rate variability, *IкB* I kappa B kinase, *IL* Interleukin, *NF-кB* nuclear Factor kappa B, *sAA* salivary alpha-amylase

To assess autonomic responses, stress-induced changes of sAA concentrations will be measured using saliva collection tubes (Salivette; Sarstedt, Nümbrecht, Germany). Furthermore, autonomic responses will be assessed via heart rate (HR) and heart rate variability (HRV) measurements (e.g., root mean square of successive differences of consecutive R–R intervals; RMSSD). To assess HPA axis activity, changes of salivary cortisol concentrations will be measured via saliva collection tubes (Salivette; Sarstedt, Nümbrecht, Germany). Peripheral immune responses to stress will be assessed by measuring changes of plasma interleukin(IL)-6 concentrations via blood sampling tubes (Monovette; Sarstedt, Nümbrecht, Germany). To determine stress-induced changes of pro- and anti-inflammatory cytokine expression rates (IL-6; IL-1β; nuclear Factor kappa B, NF-кB; I kappa B kinase, IкB), additional blood samples will be collected using tubes containing ribonucleic acid (RNA) stabilization solution (Tempus Blood RNA tube; ThermoFisher Scientific, Darmstadt, Germany).

### Experimental setting and procedure

Experimental sessions will take place on two consecutive days. On each day, participants will be scheduled between 12:30 and 15:00 to minimize the impact of circadian variations of cortisol and sAA secretion [53]. Participants will be instructed to refrain from exercising for 24 h prior to the visit, and from smoking, brushing teeth, or eating or drinking anything except water for 1 h before the visit.

After arrival at the laboratory, participants will be accommodated in a comfortable armchair and receive verbal and written instructions from trained study staff. Upon providing verbal and written informed consent regarding the collection and use of their data and biological specimens, participants will be fitted with HRV sensors (Portabiles, Erlangen, Germany). They will then be asked to provide a baseline saliva sample, followed by the initial placement of a peripheral venous catheter (Vasofix Braunüle, Braun, Melsungen, Germany). After a 45-min resting period to ensure adequate recovery from either the catheter insertion or travel to the laboratory, participants will be introduced to a modified version of the original TSST procedure [54]. During this procedure, participants will be guided to a test room and introduced to a selection committee comprising one female and one male observer wearing white lab coats. Participants will be instructed to take the role of a job applicant and deliver a 5-min speech to present themselves as the best candidate for a vacant position (i.e., their “dream job”). They will receive 5 min to prepare for their talk, after which they will be instructed to stand in front of the panel and begin their speech. Once their presentation is completed, participants will be given a mental arithmetic task for additional 5 min, in which they will be asked to serially subtract 17 from 2043 as quickly as possible. In the case of any error, they will be requested to start over. The panel will remain neutral and provide no feedback throughout the entire task. Additionally, both the 5-min speech as well as the mental arithmetic task will be audio- and videotaped for further analysis. Saliva samples will be collected immediately before (–1) as well as + 1, + 10, + 20, + 30, and + 45 min after the TSST. Plasma and RNA samples will be collected immediately before (–1) as well as + 30 and + 120 min after stress exposure. Psychological assessments will be carried out before, during, and after completion of the TSST via questionnaires (see “Psychological assessments during the experiment”).

All participants will then be randomly assigned to one of two intervention programs (intervention group 1: rumination, intervention group 2: self-compassion) or to a control condition. Participants will then receive verbal and written instructions for the upcoming task and will be asked to either engage in coping behavior (rumination vs. self-compassion) or to think neutrally about a given topic (e.g., description of everyday life) and to write down their thoughts. To check whether the experimental manipulations were successful, psychological variables will be measured repeatedly before and after each experimental condition via questionnaires (see “Psychological assessments during the experiment”). Upon completion, participants will be dismissed and reminded of the follow-up appointment (+ 1 day) and the incentive they will receive via email. With this email, participants will receive instructions to reflect on the experienced stress situation, to review and complete their notes, and to bring these notes to session 2.

The following day, participants will be scheduled at approximately the same time for experimental session 2. Procedures will closely follow those of experimental session 1, with participants being instructed to review their notes from the previous session and engage in a slightly modified version of the mental arithmetic task during the TSST (i.e., serially subtracting 13 from 2011). In contrast to experimental session 1, no further intervention will take place on this day. At the end of this session, participants will be debriefed and dismissed. To ensure quality control of the experimental procedure, regular audits will take place at intervals of 6 to 8 weeks. These audits will be conducted by a principal investigator who is independent of the data collection process. The audits will include re-training sessions for the TSST panel, as well as for staff members who interact with participants. The objective of these audits is to reinforce adherence to the protocol, address any procedural deviations, and maintain a high standard of data integrity throughout the study.

### Outcome measures

#### Primary outcomes

Primary outcomes will include self-reported measures of affect, appraisals of task-related stress, trait and state rumination, as well as trait and state self-compassion. Further primary outcomes will be response and habituation indices of biological stress measures for HPA axis, autonomic, and inflammatory responses (i.e., cortisol, sAA, HR, HRV, plasma IL-6, cytokine expression rates of IL-6, IL-1β, NF-кB, IкB). To obtain response indices, delta scores will be computed for the initial and repeated exposure to stress using peak values relative to baseline (e.g., peak cortisol values at + 1, + 10, or + 20 min post-TSST minus cortisol values at –1 min pre-TSST). To obtain habituation indices of biological stress measures, values of the second stress response will be subtracted from the first stress response values.

#### Secondary outcomes

Secondary outcomes will be psychological (e.g., emotion regulation, coping), demographic (e.g., age, gender, sex), and anthropometric (e.g., BMI) variables. Associations between primary and secondary outcomes will be analyzed in an exploratory fashion.

### Hypotheses

By increasing either ruminative or self-compassionate thoughts among healthy young adults, the intervention programs are expected to affect the regulation of occurring emotions as expressed by the responsiveness of biological systems during repeated stress exposure.

To check whether the experimental manipulations are successful on a psychological level, self-report measures will be assessed repeatedly over the course of each experimental session and are expected to change over the course of experimental sessions 1 and 2. Specifically, we hypothesize that stress will elicit changes in affect scores and stress appraisals, whereas the two intervention programs are expected to induce changes in affect, self-compassion and rumination, which will be stronger than in the control group (no intervention). Furthermore, we expect these variables to predict indicators of biological stress system activity.

- Hypothesis 1: Independent of experimental session and group, stress will elicit significant increases of negative affect and stress appraisals as well as decreases of positive affect.
- Hypothesis 2: At experimental session 1, stress-induced changes of positive and negative affect and stress appraisals will be indifferent between participants from intervention groups 1 (rumination) and 2 (self-compassion) as well as participants from the control group (no intervention).
- Hypothesis 3: At experimental session 2, stress-induced decreases of positive affect as well as increases of negative affect and stress appraisals will be strongest in participants from intervention group 1 (rumination) and lowest in participants from intervention group 2 (self-compassion).
- Hypothesis 4: Increases in state self-compassion and positive affect from pre- to post-intervention will be strongest in intervention group 2 (self-compassion), and increases in state rumination and negative affect will be strongest in intervention group 1 (rumination).

Furthermore, we expect significant responses of biological stress system activity in all experimental groups at each experimental session. Specifically, we expect responses of biological stress system activity to be similar at experimental session 1 and participants from intervention group 2 (self-compassion) to show stronger habituation of stress system activity compared to intervention group 1 (rumination) or the control group (no intervention).

- Hypothesis 5: Independent of experimental session and group, stress elicits significant responses of biological stress system activity.
- Hypothesis 6: At experimental session 1, responses of biological stress system activity will be indifferent between participants from intervention groups 1 (rumination) and 2 (self-compassion) as well as participants from the control group (no intervention).
- Hypothesis 7: At experimental session 2, responses of biological stress system activity will be strongest in participants from intervention group 1 (rumination) and lowest in participants from intervention group 2 (self-compassion).
- Hypothesis 8: Habituation of biological stress system activity will be lowest in participants from intervention group 1 (rumination) and strongest in participants from intervention group 2 (self-compassion).
- Hypothesis 9: Self-compassion and positive affect will predict lower biological responses to repeated stress, whereas rumination, negative affect, and stress appraisals will predict higher biological responses to repeated stress.
- Hypothesis 10: Self-compassion and positive affect will predict stronger habituation of biological stress system activity to repeated stress, whereas rumination, negative affect, and stress appraisals will predict lower habituation of biological stress system activity to repeated stress.

We expect these changes to occur among markers of the HPA axis, the autonomic system, as well as the inflammatory system.

Associations of biological stress system activity with other psychological variables (e.g., emotion regulation, coping) as well as demographic (e.g., age, gender, sex) and anthropometric (e.g., BMI) variables will be tested in an exploratory fashion.

### Sample handling and laboratory analysis of biological samples

Saliva samples will be stored for later analyses at –30°C after each experimental session. Upon completion of the entire data collection, the frozen salivettes will be thawed and centrifuged at 2000*g* and 20°C for 10 min. Salivary cortisol concentrations will be determined—as described by Janson and Rohleder [55]—in duplicate using a commercially available chemiluminescence immunoassay (IBL, Hamburg, Germany). Final measurements of sAA concentrations will be completed—as described by Kuras et al. [56] and Rohleder and Nater [57]—using an enzyme-kinetic method. Analogous to our previous studies [e.g., 58], peripheral immune responses to stress will be assessed via blood samples which will be centrifuged immediately at 2000*g* and 4°C. Plasma will then be aliquoted and stored for later analyses at − 80°C. IL-6 concentrations will be determined using a commercial high-sensitivity enzyme-linked immunosorbent assay (R&D Systems, Wiesbaden-Nordenstadt, Germany).

As was successfully implemented in our previous studies [e.g., 59], cytokine expression of pro- and anti-inflammatory proteins will be used to map intracellular regulation of inflammation. For this purpose, blood samples will be stored at − 80°C until RNA isolation using a commercial RNA isolation kit (Tempus Spin RNA Isolation Kit; ThermoFisher Scientific, Darmstadt, Germany). Aliquots will then be stored at − 80°C until processing. Extracted RNA concentration will be assessed with the Infinite M200pro (Tecan, Zürich, Switzerland) using NanoQuant plates™ (Tecan, Zürich, Switzerland). RNA will be translated into cDNA with the T100TH Thermal Cycler (Bio-Rad, Munich, Germany) using a commercially available High Capacity cDNA RT KIT (ThermoFisher Scientific, Darmstadt, Germany). cDNA will be diluted with nuclease-free, steam-sterilized water (Carl Roth, Karlsruhe, Germany) resulting in 35 ng cDNA per 8 µl template (reference range of ThermoFisher Scientific: 10–100ng). Real-time PCR will be performed with the ABI Prism 7000 Sequence Detection System (Applied Biosystems, Darmstadt, Germany). TaqMan™ Gene Expression Master Mix (ThermoFisher Scientific, Darmstadt, Germany) will be applied for 20 µl probe dilution per well. Cycler conditions will be applied following the manufacturer’s standard protocol. To detect the presence of complementary nucleic acids, commercially available FAM-labelled primer variants will be used (IL-6: Hs00174131_m1, IL-1β: Hs01555410_m1, RELA: Hs00153294_m1, NFKBIA: Hs00153283_m1; ThermoFisher Scientific, Darmstadt, Germany). VIC-labelled GAPDH (Primer-Limited, Hs99999905m1; ThermoFisher Scientific, Darmstadt, Germany) will be used as housekeeping gene and endogenous control. All expression levels will be determined in duplicates. Relative quantification will be assessed via measurement of fluorescence signals of FAM and VIC that accelerate proportionally to resulting PCR products. Data will be normalized against GAPDH gene expression using the ΔΔCT method (ΔΔCT = ΔΔCT target—ΔΔCT control) and expression will be normalized to the baseline for each experimental session. Upon completion of laboratory analyses, deidentified biological specimens will be preserved for potential re-analyses at the Laboratory of the Chair of Health Psychology.

### Analysis of heart rate variability

To determine autonomic responses, HR and HRV parameters will be recorded by using HRV sensors (Portabiles, Erlangen, Germany) which enable recordings of one-channel ECG signals with a 256-Hz sampling frequency. The ECG signal will be derived using dry electrodes being integrated into a chest strap. Both sensor and strap will be attached to the participant at the beginning of each experimental session to record time-dependent changes in HR and HRV using time- (RMSSD) and frequency-based (high frequency) parameters. Five-minute intervals (− 10 min pre-TSST, TSST 1, TSST 2, TSST 3, + 10 min post-TSST) will be chosen for data analysis. Data transfer will be carried out using the application “Portabiles Study Center” for Android mobile devices (Redmi 10, Xiaomi, Beijing, China).

### Randomization

Before testing, eligible patients will be randomized and stratified for sex using the Robust Randomization App (RRApp, Version 3.0.1). A principal investigator, who is independent of the enrollment and data collection, will be responsible for the randomization process. He will store the randomization lists in sealed envelopes and hand them out to staff members immediately before the experimental sessions begin. Participants will be blinded and will not know the experimental condition to which they have been assigned. Staff members will be encouraged to maintain blinding as far as possible. The actual allocation will not be disclosed to the participants, and any code breaks must be promptly reported, along with the reason for the breach. Unblinding of participants will result in study discontinuation. To assess the effectiveness of the blinding procedures, as well as to gain insight into participant perceptions of their assigned interventions, participants will be asked whether they have any suspicions about which intervention group they have been assigned to upon completion of all laboratory sessions. As the analyst is also involved in the data collection process and therefore possesses knowledge of the participants’ group assignments, no double-blinding will be implemented for the data analysis. This potential for bias will be mitigated by adhering strictly to the pre-registered analysis plan [60], which outlines the statistical methods and criteria for evaluating outcomes.

### Data preparation and statistical analysis

Data preparation and statistical analysis will be performed using IBM SPSS Statistics 26 (IBM Corp., Armonk, NY, USA). Following our pre-registered analysis plan [60], data will be screened for outliers using *z*-scores, and outliers which differ more than 3 standard deviations from the participants’ mean will be excluded from further analysis. In addition, participants will be excluded in case of elevated cortisol levels (e.g., > 12 nmol/L or *z* > 3.29) after arrival at the laboratory (i.e., at baseline) and after accommodating to the testing situation (i.e., at − 1 min relative to the TSST). Additionally, non-adherent participants whose missing values exceed 25% of the data will also be excluded from the analysis. In the next step, Kolmogorov–Smirnov tests will be conducted in order to check for normal distribution. If necessary, data will be log-, ln-, or inverse transformed to reduce skewness of biological data.

To analyze the data (i.e., to compare self-report measures as well as biological response and habituation indices between experimental groups), we will use separate analyses of covariance (ANCOVAs). Sex and BMI will be used as covariates in analyses of biological data, as these two variables have been repeatedly shown to be highly relevant for stress system activity [61, 62]. We will use the standard *p* < 0.05 criteria for determining if the ANCOVA and the post hoc test suggest that the results are significantly different from those expected if the null hypothesis were correct. The post-hoc Bonferroni test adjusts for multiple comparisons.

To investigate associations between primary psychological outcome measures and indices of biological stress measures, we will compute separate hierarchical linear regression models, regressing biological outcome measures onto psychological outcome variables. Again, sex and BMI will be included as covariates in these analyses. When examining state variables, the respective trait variable will be included as covariate in regression analyses.

We expect that certain psychological (e.g., emotion regulation, coping), demographic (e.g., age, gender, sex), and anthropometric (e.g., BMI) variables may be related to biological stress system activity. Therefore, we will test for relationships between these variables and primary outcome measures of biological stress responses in an exploratory fashion.

### Data management plan

All data will be treated in compliance with the applicable German and European data protection regulations. All data will be pseudonymized, i.e., a random numeric code will be generated and used as data identifier. This code does not allow any conclusions to be drawn about the person. Personal data will be stored separately from all other data in computer files. These files will be protected by password and accessibility will be restricted to the principal investigators.

One year after the completion of the data analysis, all personal data will be deleted. In accordance with recommendations from the DFG, deidentified data will be stored on secure file servers for 10 years. Upon completion of the study, deidentified data will be made available to other researchers via public repositories. Only mean values and group statistics will be reported in publications.

### Oversight and monitoring

Given the foundational character of the study, its low complexity and minimal risk to participants, as well as its lack of immediate impact on specific patient groups or the general public, no formal committees have been established for this study. Instead, the relevance of the study will be ensured through close collaboration with external parties (i.e., the Ethics Committee of the FAU Medical Faculty). Responsibility and organization are shared among the principal investigators and are coordinated through weekly meetings.

### Dissemination and analysis plans

Data analyses will be carried out upon completion of the entire data collection. Preliminary analyses of the data are not intended. At least one paper will be submitted to a peer-reviewed journal in the field. Pre-processed and anonymized data will be made publicly available to other researchers via public repositories. Upon completion of data collection and laboratory analyses, participants will receive individualized feedback in the form of a cortisol profile. This profile will provide insights into their personal stress responses and may indicate potential adrenal dysfunctions. Consistent with our standard practices for laboratory studies, this summary will be prepared in lay language to ensure that participants can easily interpret the results related to their health and stress responses.

## Discussion

### Summary

In summary, habituation of biological stress system activity to repeated stress exposure is considered a protective mechanism counteracting the accumulation of allostatic load [12]. However, due to the scarcity of experimental studies demonstrating that psychotherapeutic intervention programs may effectively modify the habituation capacity of biological stress system activity, it remains unclear whether stress response habituation of other health-relevant systems, including the SNS and especially the inflammatory system, may be targeted using similar intervention programs. Therefore, this study aims to address this gap in research and to investigate possible paths of action by manipulating two different coping strategies being either positively [self-compassion, 13] or negatively [rumination, 14] related to biological stress response habituation. Overall, we anticipate self-compassion to promote and rumination to impede biological stress response habituation.

### Strengths

There are several strengths of this study that contribute significantly to the field of stress research and its implications for stress-related diseases. Firstly, this is an innovative experimental study that tests short interventions capable of influencing acute stress responses in two distinct directions. By including two experimental conditions (rumination vs. self-compassion) and a control condition, along with randomized assignment, the study thoroughly examines the potential paths of action.

Secondly, our study is the first to comprehensively include subjective, endocrine, and inflammatory stress responses. This holistic approach allows for a more thorough understanding of how cognitive coping strategies can modify not only just psychological perceptions of stress but also the biological stress responses mediated by the HPA axis, autonomic nervous system, and inflammatory processes.

Thirdly, the practical implementation of short-term interventions in our study stands out as a significant advantage. More specifically, the brevity of our interventions enhances their feasibility and applicability in real-world settings. Short-term interventions are not only easier to administer but also more likely to be adopted by individuals and integrated into daily routines, which may help to broaden the potential impact of our findings.

### Risks

One potential risk of the study is that the duration of the interventions might not be sufficient to induce the desired changes in stress system reactivity and inflammatory responses. This could result in an inability to detect meaningful differences between the experimental conditions and the control condition. To minimize this risk, we will ensure that the interventions are based on evidence from our own previous studies that have shown significant effects on stress response habituation within similar timeframes.

Another potential risk is that participants might not fully adhere to the intervention protocols or engage with the cognitive coping strategies as intended, compromising the effectiveness of the interventions. Monitoring adherence during sample collection and sending detailed instructions via email to enhance engagement can help mitigate this risk.

Furthermore, although complications associated with blood drawing, such as pain, bleeding, or vein damage, are rare, they may still occur. Should any such complication arise, the participant will receive care from a research physician, be thoroughly debriefed, and subsequently dismissed from the study. In the event of a withdrawal of consent, the participant will also be debriefed and dismissed from the study.

### Limitations

One major limitation of the present study is that we will not be able to control for the effects of leukocyte trafficking, which implies that the changes observed cannot be clearly attributed to either stress-induced migration of leukocytes with activated inflammatory mechanisms into peripheral blood or the actual activation of the inflammatory cascade within the individual leukocytes [63, 64]. Future studies will need to control for effects of leukocyte trafficking using appropriate laboratory techniques ahead of RNA extraction.

Furthermore, eligible participants for the study will likely be younger than 40 years of age and generalizability of the results will be limited to some extent. However, our target sample represents a young and healthy population from which we would expect minor risk of morbidity being associated with dysregulations of biological stress systems including, for example, major depression [37], chronic fatigue [65], hypertension [66], or the metabolic syndrome [67]. Future studies should investigate whether the findings derived from this study generalize to more diverse populations, including individuals from other groups of age (e.g., above 40 years), or to individuals with existing health conditions related to stress system dysregulation. Furthermore, longitudinal studies are needed to further investigate how rumination and self-compassion might affect immune-mediated effects on physiological functions and physical health in later life.

### Implications

Overall, the findings of the planned study help to broaden our current knowledge of the biological significance of coping strategies with regard to biological adaptation to repeated or long-term stress experience. More specifically, our results will enhance the understanding of how rumination and self-compassion may affect physical health by modulating inflammatory responses to repeated stress exposure. Further avenues of research can build upon the findings to advance our understanding of coping strategies’ biological implications and enhance the development of targeted interventions for stress management and health promotion.

### Conclusions

Our study will expand our current knowledge about the modifiability of biological stress and immune response patterns that are known to play a crucial role in the pathway linking stress and disease [12]. Specifically, by increasing indices of habituation to repeated stress exposure, beneficial effects of cognitive–behavioral techniques for physical health will be made visible. Overall, the findings will have important implications, especially in the context of psychotherapeutic stress management programs.

## Trial status

The study is at the recruitment stage when this manuscript is completed. Recruitment starts in August 2024 and will be completed in September 2026. This study protocol is version 2.0, dated November 06, 2024. The end of this trial is expected in September 2027.

## Supplementary Information

Additional file 1.

Additional file 2.

Additional file 3.

## Data Availability

The final dataset will be available for the principal investigators. Upon completion of the study, the final trial dataset will be made accessible in a public repository.

## Abbreviations

ANCOVA: Analysis of covariance
BMI: Body mass index
DFG: German Research Foundation
DRKS: German Clinical Trials Register
ECG: Electrocardiogram
FAU: Friedrich-Alexander-Universität Erlangen-Nürnberg
HPA axis: Hypothalamus–pituitary adrenal axis
HR: Heart rate
HRV: Heart rate variability
IкB: I kappa B kinase
IL: Interleukin
NF-кB: Nuclear Factor kappa B
RMSSD: Root mean square of successive differences of consecutive R-R intervals
RNA: Ribonucleic acid
sAA: Salivary alpha-amylase
SNS: Sympathetic nervous system
TSST: Trier Social Stress Test
