# Supplementary material for: Interventions to modify the habituation of biological responses to repeated stress in healthy adults: a randomized controlled trial
Source: Trials. 2024 Nov 20;25:783. doi: 10.1186/s13063-024-08620-w (PMC11577917; doi:10.1186/s13063-024-08620-w)
Supplement: Supplementary file 2 — Additional file 2. [file 13063_2024_8620_MOESM2_ESM.pdf]

All items from the World Health Organization Trial Registration Data Set **(Version 1.3.1)**

Data category	Information
Primary Registry and Trial Identifying Number	German Clinical Trials Register (DRKS) DRKS00034790
Date of Registration in Primary Registry	August 12, 2024
Secondary Identifying Numbers	Open Science Framework (OSF) https://doi.org/10.17605/OSF.IO/XC4F6
Source(s) of Monetary or Material Support	Deutsche Forschungsgemeinschaft (DFG)<br>Kennedyallee 40<br>53175 Bonn<br>Germany
Primary Sponsor	FAU Erlangen-Nürnberg, Lehrstuhl für Gesundheitspsychologie<br>91052 Erlangen<br>Germany
Secondary Sponsor(s)	Not Applicable
Contact for Public Queries	Johanna Janson-Schmitt, johanna.janson-schmitt@fau.de
Contact for Scientific Queries	Johanna Janson-Schmitt, johanna.janson-schmitt@fau.de
Public Title	Modification of Biological Stress Response Patterns through Experimental Manipulation of Cognitive Coping Strategies
Scientific Title	Modification of Biological Stress Response Patterns through Experimental Manipulation of Cognitive Coping Strategies
Countries of Recruitment	Germany
Health Condition(s) or Problem(s) Studied	Stress response patterns of healthy participants
Intervention(s)	Arm 1: Condition 1 will encompass a rumination intervention, in which participants will be instructed to engage in ruminative thinking and write down their thoughts following the initial stress exposure.<br>Arm 2: Condition 2 will encompass a self-compassion intervention, in which participants will be instructed to engage in self-compassion and write down their thoughts following the initial stress exposure.<br>Arm 3: Condition 3 encompasses an active control group, in which participants will be instructed to think neutrally about a specified topic (e.g., description of everyday life), and to write down their thoughts.
Key Inclusion and Exclusion Criteria	Sex: All<br>Minimum Age: 18 Years<br>Maximum Age: no maximum age
	Inclusion criteria: 1) 18 years or older, 2) living in the greater area of Nuremberg (Germany), 3) female participants: luteal phase of their menstrual cycle, 4) willingness to participate in the study and to provide biological samples
	Exclusion criteria: 1) younger than 18 years of age, 2) presence of depressive or social anxiety symptomatology, 3) presence of acute and/or chronic somatic diseases, 4) medication intake (e.g., beta blocker, glucocorticoids, anti-depressants), with the exception of hormonal contraceptives in women, 5) Body Mass Index (BMI) below 18 or above 30 kg/m^2, 6) smoking (> 10 cigarettes/week), 7) receiving psychotherapeutic treatment at study entry, 8) previous experience with the stress protocol, 9) being an employee of Friedrich-Alexander-Universität Erlangen-Nürnberg (FAU)
Study Type	Interventional

	Allocation: Randomized controlled study
	Primary purpose: Basic research/physiological study
Date of First Enrollment	September 01, 2024
Sample Size	120
Recruitment Status	Recruiting
Primary Outcome(s)	Before and after the first and second stress exposures, as well as before and after the intervention, self-reported measures will be assessed: affect (Positive and Negative Affect Schedule, PANAS), task-related stress (Short Stress State Questionnaire, SSSQ), trait and state rumination (Response Style Questionnaire, RSQ; Brief State Rumination Inventory, BSRI), and trait and state self-compassion (Self Compassion Scale, SCS; State Self-Compassion Scale Short Form, SSCS-S).<br><br>During the first and second stress exposures, biological stress and inflammatory responses will be assessed. To measure stress-induced activation of the HPA axis (cortisol), as well as autonomic (heart rate, heart rate variability, alpha amylase) and inflammatory (plasma IL-6, gene expression rates of pro- and anti-inflammatory cytokines) stress responses, repeated saliva (baseline, -1, +1, +10, +20, +30, +45 minutes relative to TSST) and blood samples (-1, +30, +120 minutes relative to TSST) will be collected, along with continuous heart rate monitoring.<br>To obtain response indices, delta scores will be computed for the initial and repeated exposure to stress using peak values relative to baseline (e.g., peak cortisol values at +1, +10, or +20 minutes post-TSST minus cortisol values at -1 minutes pre-TSST). To obtain habituation indices of biological stress measures, values of the second stress response will be subtracted from the first stress response values.
Key Secondary Outcomes	Secondary outcomes will be psychological (e.g., emotion regulation, coping), demographic (e.g., age, gender, sex), and anthropometric (e.g., BMI) variables. Associations between primary and secondary outcomes will be analyzed in an exploratory fashion.
Ethics Review	Ethik-Kommission der Friedrich-Alexander-Universität Erlangen-Nürnberg<br>Krankenhausstr. 12<br>91054 Erlangen<br>Germany<br>+40-9131-8522270<br>ethikkommission@fau.de<br>http://www.ethikkommission.fau.de
	Date of ethics committee application: January 19, 2021<br>Ethics committee number: 10_21 B
	Vote of the Ethics Committee: Approved<br>Date of the vote: February 09, 2021
Completion date	September 30, 2026
Summary Results	Not Applicable
IPD sharing statement	
