# Supplementary material for: Interventions to modify the habituation of biological responses to repeated stress in healthy adults: a randomized controlled trial
Source: Trials. 2024 Nov 20;25:783. doi: 10.1186/s13063-024-08620-w (PMC11577917; doi:10.1186/s13063-024-08620-w)
Supplement: Supplementary file 3 — Additional file 3. [file 13063_2024_8620_MOESM3_ESM.pdf]

Participation information for the MODSTR study

Dear participant,

We are pleased that you are interested in the MODSTR project at the Chair of Health Psychology at Friedrich-Alexander-University Erlangen-Nürnberg and are potentially willing to participate in the study.

We would like to inform you in advance about the goals and procedures of the study. Please read this information letter carefully. If you have any questions, do not hesitate to contact us.

1. Purpose of the Research Project

The aim of this research project is to investigate whether the human body's stress response can be influenced through mental exercises. To achieve this, we plan to expose healthy participants to an established stress test lasting about 15 minutes and a non-stress-inducing exercise of 10 minutes duration in our lab. We will measure the body's stress response using traditional methods before, during, and after these activities. This investigation will take place over two consecutive afternoons, with a total of three hours per day including preparation and follow-up. The exact procedure is described in the following text.

2. Study Procedure

The study is divided into two parts: The first part involves collecting data about you through an online questionnaire. In the second part, you will be asked to come to our lab on two consecutive afternoons.

Part 1: To get to know you better, we ask you to complete an online questionnaire beforehand. This questionnaire will gather information about your current health status and how you handle stress. Answering the questions will take a maximum of one hour and poses no risk to you.

Part 2: After completing our questionnaire, we will schedule two in-person appointments in our lab (each lasting about 3 hours). On both days, you will be asked to provide a total of 7 saliva samples (1.1 ± 0.3 ml each) and 3 blood samples (3 x 9 ml each) and participate in a 15-minute psychosocial stress test. This test includes two different mental tasks that must be solved within a given time. Video recording will take place during this test. Additionally, we will record your heart rate and heart rate variability using a portable ECG device.

We will also take one-time measurements of your body weight, height, and body fat percentage. To get a comprehensive picture of you, we also ask you to complete 5 questionnaires (duration: about 20 minutes).

On the first day, we also ask you to participate in a non-stress-inducing exercise lasting 10 minutes. After the first in-person appointment, we will remind you by email of the follow-up appointment (+1 day). At that time, we ask you to perform the non-stress-inducing exercise again from home.

3. Use of Study Materials

All blood and saliva samples will be analyzed in an internal lab to determine hormone and inflammatory marker concentrations. All blood and saliva samples will be destroyed by the Chair of Health Psychology at Friedrich-Alexander-University Erlangen-Nürnberg after the laboratory analyses are completed.

All data collected during the study (psychometric data, health status, blood and saliva analysis results, heart rate and heart rate variability recordings, video recordings) will be deleted after 10 years. The deletion of personal data will occur no later than 3 years.

4. Data Protection and Anonymity

Study results and biological materials will be pseudonymized as follows: Before starting your participation, you will receive a four-digit random code by email, which you should provide at the beginning of the online screening as a participant identifier. This code, along with the participant's name, will be recorded in a coding list. This list is kept in a locked cabinet and is accessible only to the study leaders. At the start of the subsequent lab examination, you will also be assigned a continuous participant number, which will be noted in the coding list and used to label the biological materials. Due to the pseudonymized identification (four-digit random code + continuous participant number), no personal data will be visible in the later study results.

The pseudonymized data and results collected in the research project will be stored for subsequent statistical analyses and used solely for scientific purposes.

After the study is completed, no individual or personal data will be disclosed – only group-related data will be published in publications. The fully anonymized data from this study will be made available as open data on the internet in a data archive, following the recommendations of the German Research Foundation (DFG) and the German Society for Psychology (DGPs) for quality assurance in research.

5. Voluntariness

Participation in the MODSTR study is voluntary, meaning you can withdraw from the study at any time without providing reasons.

For participating in each of the two in-person appointments, you will receive compensation of €50.00 or a credit of 3 participant hours.

You may withdraw consent for data storage until the end of data collection without facing any disadvantages. However, you will not receive any compensation in this case. Compensation will be provided only after the completion of the second in-person appointment.

6. Benefits of your Participation

By participating, you are making a significant contribution to stress research. The findings may be used to assess the risk of stress-related diseases such as cancer, cardiovascular diseases, and Alzheimer's Disease.

7. Risks

There are no fundamental risks associated with your participation. However, we want to inform you about the following:

It is possible that participants who are anxious may feel temporarily uncomfortable during the stress test. All non-invasive procedures (saliva sampling, wearing heart rate monitors and straps) are harmless.

Invasive procedures are performed only by trained medical professionals, so no complications are expected during or after blood sampling. Potential risks of blood sampling include swelling, bleeding, infections, accidental arterial puncture, or, in rare cases, nerve injury. To minimize potential complications, you will be seated comfortably during blood sampling and asked to maintain your position to avoid dizziness.

All blood samples will be taken using modern peripheral venous catheters, requiring only one puncture. After removing the needle, a plastic tube will remain in your arm, through which further blood samples will be taken. You can move your arm safely during this time. You will be released only after the cannula has been removed by the doctor and no further follow-up treatment is required.

We want to emphasize once again that you can withdraw from the study at any time without providing reasons.

Thank you for your participation.

Prof. Dr. Nicolas Rohleder Dr. Johanna Janson-Schmitt

Friedrich-Alexander-Universität Erlangen-Nürnberg
Institute of Psychology
Chair of Health Psychology – Laboratory
Fürther Straße 248
90429 Nuremberg

Phone: +49 911 5302-96627
Email: ifp-gesundheitspsychologie-jj@fau.de
http://www.gesundheitspsychologie.phil.fau.de

Friedrich-Alexander-Universität
Philosophische Fakultät und
Fachbereich Theologie

FAU

Consent form for Participation in the MODSTR Study

I have been thoroughly and clearly informed by the investigator about the purpose, procedure, significance of the study, as well as the potential benefits and risks associated with it.

Furthermore, I have read and understood the text of the patient information. All my questions have been answered to my satisfaction.

I have had sufficient time to reconsider and freely make my decision to participate in the study.

My following declarations are limited to what has been explained to me in the written participant information or in the oral explanation.

I am aware that I can withdraw my consent to participate in the study at any time and without providing reasons (orally or in writing), without facing any disadvantages.

I am aware that the data and research results collected within the scope of the research project will be stored in an anonymized form in a publicly accessible archive and used exclusively for scientific purposes.

I hereby consent to participate in the above-mentioned study.

☐ I consent to participate in the study

☐ I **do not** consent to participate in the study

For my participation in the study, I will receive a compensation of €50.00, or the duration of the examination will be credited to me as 3.00 participant hours.

I choose:
(please check)

a) ☐ the compensation of €50,00

b) ☐ credit of 3.00 participant hours

Date ____________ Name ______________________ Signature ______________

Consent Form for Scientific Use of Blood or Saliva Samples and Personal Data

Dear Study Participant,

We request your consent for the scientific use of your blood or saliva samples and your personal data, as explained to you in the participant information.

MODSTR

Chair of Health Psychology - Laboratory

Fürther Straβe 248, 3rd Floor

90431 Nuremberg

Study Directors: Prof. Dr. Nicolas Rohleder
nicolas.rohleder@fau.de

Dr. Johanna Janson-Schmitt
johanna.janson-schmitt@fau.de

A. General Information

I have been informed by the study director about the purpose, procedure, significance of the study, as well as the potential benefits and risks associated with it.

I have read the written participant information. All my questions have been answered to my satisfaction.

I have had sufficient time to consider and make my decision regarding participation in the study freely.

My following statements apply only to the extent that they have been outlined to me in the written participant information or explained verbally.

My following statements authorize and obligate the institution mentioned above.

B. Consent to the Collection and Use of Blood or Saliva Samples

(Please check applicable boxes)

B 1 Use of Study Materials

I consent to the collection of blood or saliva samples and hereby transfer the collected blood or saliva samples to the institution named above. I agree that the blood or saliva samples, under the responsibility of the named institution, will be used in encrypted form (i.e., so that identification with my person is only possible through a reference list)

☐ for studies with the research question specified above.

☐ I agree that I may be contacted by the institution named above, if possible, to give my consent for the use of my blood or saliva samples in future studies.

or

☐ I request that my blood or saliva samples be destroyed after the completion of the mentioned studies.

or

☐ for studies with all scientifically relevant research questions.

B 2 Information about Study Results

t is possible that the study may yield results indicating, for example, an increased release of the hormone cortisol from the adrenal glands or transient ECG changes (e.g., heart arrhythmias).

However, the saliva and blood values measured by the institution named above, as well as the ECG recordings, do not have medical diagnostic quality and will only be available with a significant delay (weeks to months after participation in the study). Nonetheless, the results require further diagnostic evaluation by a general practitioner or specialist.

I am aware that knowledge of or suspicion of an underlying serious illness may also have disadvantages and that further investigations may follow, with the result that the findings may not indicate a medical condition.

☐ If the study yields results that are of immediate importance to me or my immediate family members, I would like to be informed.

or

☐ I agree that I will not receive individual feedback on the results of the study.

B 3 Compensation

I am aware that for the provision of my blood and saliva samples, I will receive either

☐ a compensation of €50,00

or

☐ a credit of 3.00 participant hours.

I am aware that I have no claims to any remuneration, royalties, or any other financial benefits or profits that may be derived from research using my blood or saliva samples.

B 4 Revocation of Consent for Sample Use

I understand that I can revoke my consent for the use of my blood or saliva samples at any time and without providing reasons to the above-mentioned institution/person, and that this will not affect my subsequent medical treatment. In the event of revocation, I agree that my blood or saliva samples will be kept for control purposes. However, I have the right to request their destruction, provided that no legal regulations prevent their destruction.

I am aware that destruction of the blood or saliva samples upon my request may not be possible if the samples have been anonymized to the extent that linking them to me would require an disproportionate amount of time, cost, and effort.

C. Data Protection Consent Declaration

(Please check as applicable)

I consent to the data concerning me (including particularly information in questionnaires) being stored and processed in encrypted form under the responsibility of the institution mentioned above:

☐ for studies related to the question mentioned above.

and/or

☐ for studies concerning the following scientifically relevant research questions: health psychology research.

Note:

According to Art. 6 Para. 2 No. 3 c BayDSG (Bavarian Data Protection Act), your data can be used for scientific or historical research without additional consent if the scientific or historical interest in conducting the research project significantly outweighs your interest in excluding the purpose change and the research purpose cannot be achieved otherwise or only with disproportionate effort.

☐ I agree to this regulation.

Access by Third Parties

☐ I consent to individuals involved in data collection for theses being able to view my study data.

☐ I agree to the transfer of my data in encrypted, pseudonymized form to students writing their theses as part of the project.

Revocation of Consent to Data Use

I am aware that I can withdraw my consent to the use of my data at any time and without giving reasons to the institution or person mentioned at the beginning and that this will not affect any further medical treatment.

☐ In the event of withdrawal, I agree that my data will continue to be stored for control purposes. However, I have the right to request its deletion unless legal provisions oppose it.

Data processing remains lawful until withdrawal.

I understand that if my data is stored anonymously, it cannot be deleted at my request.

Privacy Notice

A. General Information

a. Names and Contact Information oft he Responsible Persons:

   Project Management:

   Prof. Dr. Nicolas Rohleder, nicolas.rohleder@fau.de
   Friedrich-Alexander-Universität Erlangen-Nürnberg
   Chair of Health Psychology
   Nägelsbachstr. 49a; 3rd Floor, 91052 Erlangen
   http://www.gesundheitspsychologie.phil.fau.de/
   Phone: +49 9131 85-20887 (Secretary's Office)

   Dr. Johanna Janson-Schmitt, johanna.janson-schmitt@fau.de
   Friedrich-Alexander-Universität Erlangen-Nürnberg
   Chair of Health Psychology
   Nägelsbachstr. 49b; 3rd Floor, 91052 Erlangen
   http://www.gesundheitspsychologie.phil.fau.de/
   Phone: +49 9131 85-20884

b. Contact Information of the Data Protection Officer:

   Martin Neidiger, martin.neidiger@fau.de
   Friedrich-Alexander-Universität Erlangen-Nürnberg
   Data Protection Officer of Friedrich-Alexander-Universität Erlangen-Nürnberg
   Schloßplatz 4, 91054 Erlangen
   Phone: +49 9131 85-20832

c. Legal Basis for Data Processing: Your consent

d. Duration of Storage: 10 years

e. Right to Complain: You may contact the Bavarian State Commissioner for Data Protection if you believe that the processing of your personal data is unlawful:

   The Bavarian State Commissioner for Data Protection (BayLfD)
   Prof. Dr. Thomas Petri
   P.O. Box 22 12 19
   80502 Munich

B. General Rights

The right to deletion and to be "forgotten" is restricted to the extent that your data is necessary for scientific research.

Further details are available here:

1. **Right to Deletion:**

   You have the right to request the immediate deletion of personal data concerning you from the data controller, and the data controller is obliged to delete personal data immediately if one of the following reasons applies:

   a) The personal data are no longer necessary for the purpose for which they were collected or otherwise processed.

   b) You withdraw your consent on which the processing is based, and there is no other legal basis for the processing.

   c) The personal data have been processed unlawfully.

   ou do not have the right to deletion if your data are required for scientific research and deletion would likely make it impossible or seriously impair the achievement of the purposes of this processing,

   or

   if the processing is necessary for the establishment, exercise, or defense of legal claims

2. **Notification Obligation in Connection with the Correction or Deletion of Personal Data or the Restriction of Processing:**

   The data controller must inform all recipients to whom personal data have been disclosed of any correction or deletion of personal data or any restriction of processing, unless this proves impossible or involves a disproportionate effort. The data controller will inform you of these recipients upon request.

   The right to data portability is restricted or excluded if the research is in the public interest or the data constitute a trade secret.

   Further details are available here:

3. Right to Data Portability:

a) You have the right to receive the personal data concerning you, which you have provided to a controller, in a structured, commonly used, and machine-readable format. You also have the right to transmit this data to another controller without hindrance from the controller to whom the personal data was provided, provided that the processing is carried out by automated means.

b) In exercising your right to data portability as per paragraph a), you have the right to have the personal data transmitted directly from one controller to another, where technically feasible.

c) The exercise of the right to data portability does not affect the right to erasure of data. This right does not apply to processing necessary for the performance of a task carried out in the public interest or in the exercise of official authority vested in the controller.

d) The right under paragraph 2 must not adversely affect the rights and freedoms of other persons.

4. If personal data is transferred to a third country or an international organization, you have the right to be informed about the appropriate safeguards in accordance with Article 46 GDPR in relation to the transfer.

Note: The research conducted in this study is in the public interest. Therefore, you may not exercise the right to data portability.

C. Rights Restricted by Research Purpose

The rights to rectification, restriction of processing, and information are excluded if exercising these rights is likely to make it impossible or seriously impair the achievement of the research purpose, and the restriction is necessary for the fulfillment of the research purpose.

Here you can find more details:

As a data subject, you have the following rights, provided these rights do not make it impossible or seriously impair the realization of the research purpose and the restriction is necessary for achieving the research purpose:

1. **Right to Rectification:**

   You have the right to request the controller to correct inaccurate personal data concerning you without delay. Taking into account the purposes of the processing, you also have the right to request the completion of incomplete personal data, including by means of a supplementary statement.

2. **Right to Restriction of Processing:**

   You have the right to request the controller to restrict processing when one of the following conditions applies:

   a) The accuracy of the personal data is contested by you. In this case, the restriction of processing can be requested for a duration that allows the controller to verify the accuracy of the personal data;

   b) The processing is unlawful, and you oppose the deletion of the personal data and instead request the restriction of its use;

   c) The controller no longer needs the personal data for processing purposes, but you need it for the establishment, exercise, or defense of legal claims.

   When processing is restricted, the personal data—apart from its storage—may only be processed with your consent or for the establishment, exercise, or defense of legal claims, or for the protection of the rights of another natural or legal person, or for reasons of significant public interest of the Union or a Member State.

   If you have obtained a restriction of processing, you will be informed by the controller before the restriction is lifted.

3. Rights to Information:

You have the right to request confirmation from the controller as to whether personal data concerning you is being processed; if this is the case, you have the right to obtain information about this personal data and the following details:

a) The purposes of the processing;

b) The categories of personal data being processed;

c) The recipients or categories of recipients to whom the personal data has been disclosed or will be disclosed, especially if recipients are in third countries or international organizations;

d) If possible, the planned duration for which the personal data will be stored, or, if not possible, the criteria used to determine this duration;

e) The existence of the right to lodge a complaint with a supervisory authority;

f) You have the right to obtain a copy of the personal data undergoing processing from the controller. For any further copies you request, the controller may charge a reasonable fee based on administrative costs. If you request the information electronically, it must be provided in a commonly used electronic format unless you specify otherwise.

The right to obtain a copy must not adversely affect the rights and freedoms of other individuals.

__________ Date   ____________________ Name   ______________ Signature

Consent Form for Audio and Video Recordings

I have been informed that a video recording will be made as part of this study.

I understand that the recording and evaluation of the video will be pseudonymized, meaning that a four-digit random code and a sequential participant number will be used instead of my name, and that a coding list exists on paper which connects my name with the four-digit random code and sequential participant number. The coding list is stored in a locked cabinet—accessible only to the study directors—and will be deleted after the data collection is completed. There is no likelihood that anyone involved in data evaluation will recognize me. For this reason, all individuals involved in the evaluation are bound by absolute confidentiality and are not permitted to disclose confidential information to third parties under any circumstances.

I am aware that I can withdraw my consent for the storage of these data without any disadvantages. The video recordings will be kept in a locked cabinet. I have been informed that I can request the deletion of my recordings at any time as long as the coding list exists. However, the recordings will be destroyed in any case after the evaluation is complete, but at the latest after ten years.

I agree with the described handling of the collected recordings.

The consent for the video recording is voluntary. I can revoke this consent at any time. In the case of refusal or withdrawal, I will not incur any costs or other disadvantages; participation in the study is still possible.

I am informed that my data will remain in pseudonymized form (coding list) until the final completion of data collection and/or evaluation and only the study management will have access to it. My personal data will be deleted after a maximum of 3 years. Until then, I can request information about my personal data and demand the deletion of this data at any time.

I have had sufficient time to make a decision. I have read and understood everything and hereby consent to being recorded on video.

☐ *Yes* ☐ *No*

Date	Name	Signature
